# Supplementary material for: Real time monitoring of hydrogenotrophic methanogenesis under deep saline aquifers conditions
Source: J Biol Eng. 2026 Jan 20;20:33. doi: 10.1186/s13036-025-00584-y (PMC12905862; doi:10.1186/s13036-025-00584-y)
Supplement: Supplementary file 2 — Supplementary Material 2 [file 13036_2025_584_MOESM2_ESM.docx]

**ESI-2**

Real time monitoring of hydrogenotrophic methanogenesis under deep saline aquifers conditions

Emeline Vidal^1^, Anaïs Cario^1^, Mathilda Jouvin^1^, Maïder Abadie^1^, Olivier Nguyen^1^, Arnaud Erriguible^1,2^_,_ Anthony Ranchou-Peyruse^3*^, Samuel Marre^1*^

^1^ CNRS, Univ. Bordeaux, Bordeaux INP, ICMCB, F-33600, Pessac Cedex, France

^2^ CNRS, Univ. Bordeaux, Bordeaux INP, I2M, site ENSCPB, 16 avenue Pey-Berland, Pessac Cedex, France

^3^ Universite de Pau et Pays de l’Adour, E2S UPPA, CNRS, IPREM, Pau, 64000, France

**Evolution of the pH of the AGW medium as a function of the H_2_/CO_2_ partial pressure**

The pH of the AGW growth medium was measured with a pHmeter (HANNA Instruments, pH HI 2210 pH meter) once the system is decompressed.

Although the medium was supplemented with a buffer (HEPES, 120 mM), the pH is decreasing down to 5.7 when the pressure is reaching p(H_2_/CO_2_) = 100 bar, while it is initially set at 6.8.

However, the buffer is efficient as the pH can be stabilized at this value and remain between 5.5 and 6 for all of the measured conditions (Figure ESI2-1). Despite these slightly acidic conditions, it remains in the viable zone for our selected strain, suggesting that it should be able to proliferate and has an active metabolism.


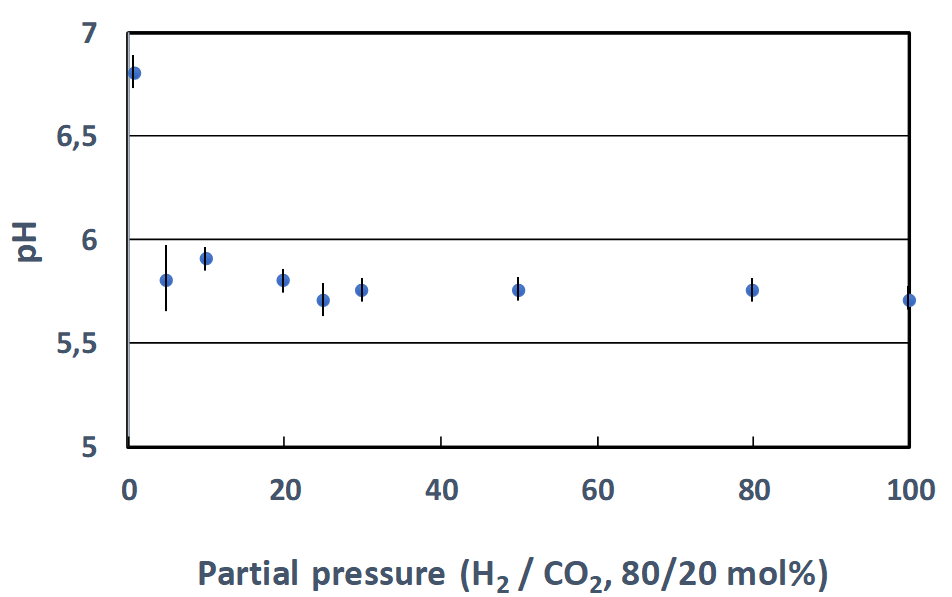


**Figure ESI2-1.** Ex situ measurement of pH value of AGW media after pressurising it with H_2_ / CO_2_ (4:1).
