# Supplementary material for: Real time monitoring of hydrogenotrophic methanogenesis under deep saline aquifers conditions
Source: J Biol Eng. 2026 Jan 20;20:33. doi: 10.1186/s13036-025-00584-y (PMC12905862; doi:10.1186/s13036-025-00584-y)

**ESI-3**

Real time monitoring of hydrogenotrophic methanogenesis under deep saline aquifers conditions

Emeline Vidal^1^, Anaïs Cario^1^, Mathilda Jouvin^1^, Maïder Abadie^1^, Olivier Nguyen^1^, Arnaud Erriguible^1,2^_,_ Anthony Ranchou-Peyruse^3*^, Samuel Marre^1*^

^1^ CNRS, Univ. Bordeaux, Bordeaux INP, ICMCB, F-33600, Pessac Cedex, France

^2^ CNRS, Univ. Bordeaux, Bordeaux INP, I2M, site ENSCPB, 16 avenue Pey-Berland, Pessac Cedex, France

^3^ Universite de Pau et Pays de l’Adour, E2S UPPA, CNRS, IPREM, Pau, 64000, France

**Calculated values of dissolved gases under the experimental conditions of Dupraz *et al.***

**Table 2.** Calculated dissolved H_2_ and CO_2_ in the experimental conditions of Dupraz et al.: Dupraz, S.; Fabbri, A.; Joulian, C.; Dictor, M.-C.; Battaglia-Brunet, F.; Menez, B. Impact of CO2 Concentration on Autotrophic Metabolisms and Carbon Fate in Saline Aquifers–A Case Study. Geochim. Cosmochim. Acta 2013, 119, 61–76.


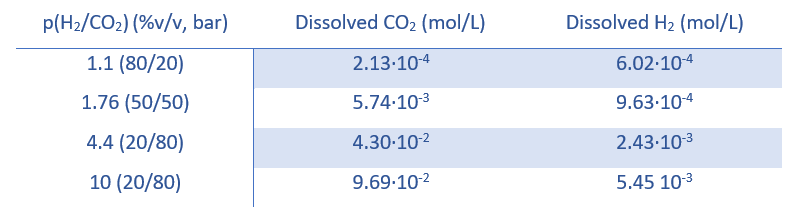

Supplement: Supplementary file 3 — Supplementary Material 3 [file 13036_2025_584_MOESM3_ESM.docx]
